# Protective Effect of Oxytocin on Ventilator-Induced Lung Injury Through NLRP3-Mediated Pathways

**DOI:** 10.3389/fphar.2021.722907

**Published:** 2021-10-18

**Authors:** Xiaomei Yang, Xiaona An, Cheng Wang, Feng Gao, Yicheng Lin, Wenjing Chen, Qiming Deng, Dongsheng Xu, Shengqiang Li, Peng Zhang, Baozhu Sun, Yuedong Hou, Jianbo Wu

**Affiliations:** ^1^ Department of Anesthesiology, Qilu Hospital, Cheeloo College of Medicine, Shandong University, Jinan, China; ^2^ School of Medicine, Cheeloo College of medicine, Shandong University, Jinan, China; ^3^ Department of Cardiology, The Key Laboratory of Cardiovascular Remodeling and Function Research, Chinese Ministry of Education, Chinese National Health Commission and Chinese Academy of Medical Sciences, The State and Shandong Province Joint Key Laboratory of Translational Cardiovascular Medicine, Qilu Hospital, Cheeloo College of Medicine, Shandong University, Jinan, China; ^4^ Department of Anesthesiology, Zibo Central Hospital, Shandong University, Zibo, China; ^5^ Shandong Qianfoshan Hospital, Cheeloo College of Medicine, Shandong University, Jinan, China; ^6^ Department of Cardiology, Affiliated Hospital of Shandong University of Traditional Chinese Medicine, Jinan, China; ^7^ Department of Kidney Transplantation, The Second Hospital, Shandong University, Jinan, China

**Keywords:** ventilator-induced lung injury (VILI), oxytocin (OT), pyroptosis, NLRP3, inflammasome

## Abstract

Mechanical ventilation is an indispensable life-support treatment for acute respiratory failure in critically ill patients, which is generally believed to involve uncontrolled inflammatory responses. Oxytocin (OT) has been reported to be effective in animal models of acute lung injury. However, it is not clear whether Oxytocin has a protective effect on ventilator-induced lung injury (VILI). Therefore, in this study, we aimed to determine whether OT can attenuate VILI and explore the possible mechanism of this protection. To this end, a mouse VILI model was employed. Mice were pretreated with OT 30 min before the intraperitoneal injection of saline or nigericin and ventilation for 4 h, after which they were euthanized. Pathological changes, lung wet/dry (W/D) weight ratio, myeloperoxidase (MPO) activity, the levels of inflammatory cytokines [i.e., interleukin (IL)-1β, IL-6, and IL-18] in lung tissues and bronchoalveolar lavage fluid (BALF), and expression of NLRP3, Toll-like receptor 4 (TLR4), caspase-1, nuclear factor (NF)-κB, and GSDMD in lung tissues were measured. OT treatment could reduce pathological injury, the W/D ratio, and MPO activity in VILI mice. Our data also indicated that OT administration alleviated the expression of TLR4/My-D88 and the activation of NF-κB, NLRP3, and caspase-1 in lung tissues from the VILI mice model. Furthermore, OT also decreased the levels of IL-1β, IL-6, and IL-18 in the bronchoalveolar lavage fluid. Moreover, the OT administration may alleviate the activation of GSDMD partially through its effects on the NLRP3-mediated pathway. Collectively, OT exerted a beneficial effect on VILI by downregulating TLR4-and NLRP3-mediated inflammatory pathways.

## 1 Introduction

Mechanical ventilation (MV), as an essential life-support treatment for the acute respiratory failure in critically ill patients (Goligher et al., 2015; Lee et al., 2020), may lead to local and systemic inflammatory responses due to lung over-distension, which may cause the occurrence and development of ventilator-induced lung injury (VILI) (Slutsky and Ranieri, 2013). Uncontrolled inflammatory responses are generally presumed to play critical roles in VILI (Cressoni et al., 2016). Despite low tidal volumes and maintenance of positive-end expiratory pressure, there is still high mortality in critically ill patients (Park et al., 2015). Thus, studies exploring effective treatments for VILI are essential.

Toll-like receptor 4 (TLR4) primarily was thought to be the sensor of pathogen-associated molecular patterns (PAMPs) (Takeuchi et al., 1999) that acted by activating nuclear factor kappa-light-chain-enhancer in activated B cells (NF-κB) and inducing the release of inflammatory chemokines and cytokines (Hu et al., 2010; Guijarro-Muñoz et al., 2014; Hongli et al., 2018). The NLRP3 inflammasome, which is a NOD-like receptor located in the cytoplasm, It is composed of apoptosis-related speckle-like protein (ASC), caspase-1, and NLRP3.

Oxidative stress was recently shown to activate the NLRP3 inflammatory corpuscles and caspase-1, which causes pyroptosis (Wu et al., 2018). In recent years, functions of other inflammatory caspases and gasdermin D (GSDMD), which is the critical substrate and the direct mediator of pyroptosis, have been discovered to provide insights into the mechanism of pyroptosis (Pandeya et al., 2019; Xia 2020). Moreover, active caspase-1 plays an essential role in cleaving GSDMD, a member of the gasdermin family, to induce pyroptosis (Yang et al., 2016; Wu et al., 2018). In addition, GSDMD, as a family of pore-forming proteins involved in the immune response, has been recently described as participating in the immune regulation in various inflammatory disease models, including inflammatory bowel disease (IBD), sepsis, and autoimmune diseases (Toldo et al., 2015). However, researchers have not examined whether inflammasome-activated GSDMD contributed to the progression of VILI-induced inflammation. GSDMD was identified by two independent screening approaches as a critical effector of pyroptosis (Man and Kanneganti 2015; Toldo et al., 2015; He et al., 2016).

Oxytocin (OT), a 9-amino acid neuropeptide, is synthesized in a limited number of discrete brain regions, including the supraoptic, paraventricular (PVN), and accessory nuclei of the hypothalamus (Althammer and Grinevich, 2017). In addition to its typical effects, OT was recently shown to play a role in the respiratory system as well, and evidence showed that asthma worsened in approximately 30% of pregnant women (Chanez et al., 2007; Almqvist et al., 2008). In pathological conditions, the anti-inflammatory properties of OT have attracted severe concern. In addition, in response to inflammatory stimuli, macrophage OT receptor (OTR) expression is dramatically upregulated, suggesting that OTR may contribute to OT’s anti-inflammatory effect (Szeto et al., 2017). Meanwhile, L-368,899, a selective antagonist of the oxytocin receptor, could weaken OT’s effects. As shown in our previous study, OT reduced lipopolysaccharide (LPS)-induced injury in the lungs, and the levels of NLRP3 inflammatory factors, TLR4, IL-1β, IL-18, and IL-6, the effects of OT could be weakened by pretreatment with L-368,899 (An et al., 2019). Moreover, nigericin, the NLRP3 activator, was applied to further explain the potential anti-inflammatory mechanism of OT (Humayun et al., 2019). However, it is not clear whether OT can exert protective anti-inflammatory effect during MV.

**FIGURE 1:**
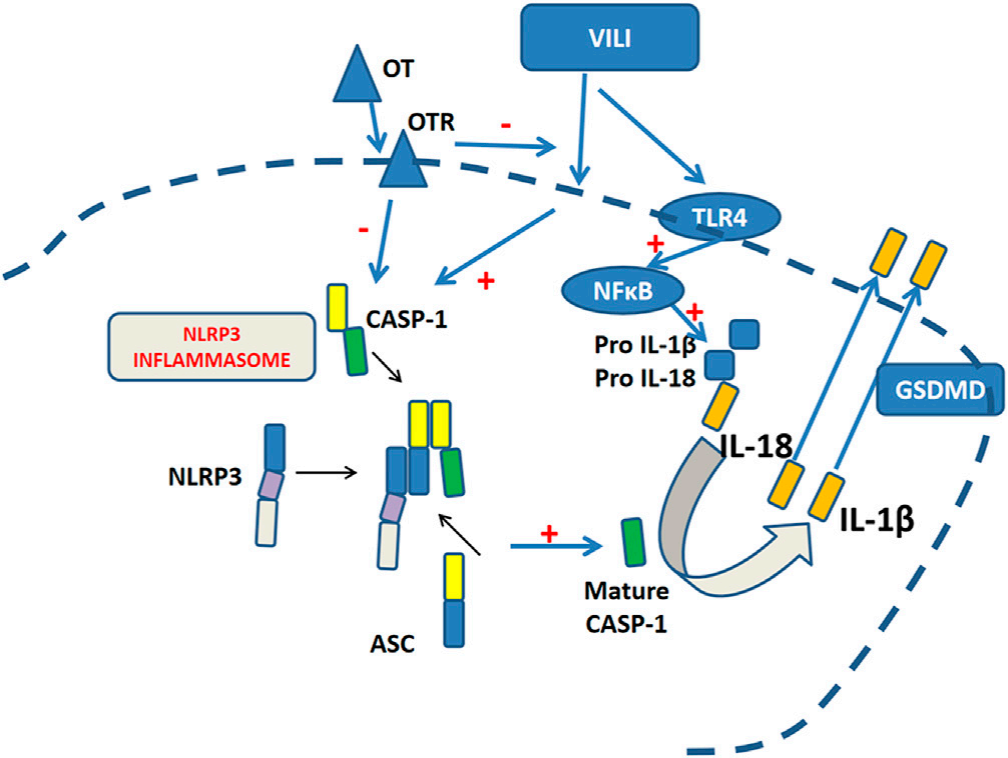
Oxytocin protects against ventilation-induced lung injury through NLRP3- mediated inflammatory pathways.

The present study aimed to explore OT’s possible effect on VILI and identify the underlying molecular mechanisms. Our present study found that OT protected against VILI through NLRP3 pathways (Figure 1).

## 2 Materials and Methods

### 2.1 Animals

Male C57BL/6 mice, ages 6–8 weeks and weighting approximately 20–22 g were obtained from the Center of Experimental Animal at the Shandong University (Jinan, China). The mice were housed in specific conditions, with laboratory temperature (approximately 22–27°C) and relative humidity (40–60%) on a 12 dark/12 h light cycle, and received unlimited access to food and water. All experimental animal protocols were approved by Experimental Animals of Shandong University.

### 2.2 Animal Model of VILI and Experimental Design

#### 2.2.1 Part One

Male C57BL/6 mice were randomly divided into five groups and all drugs were administered intraperitoneally (ip): 1) Control group [90 min saline +30 min saline + MV (7 ml/kg, 120 breaths/min)]; 2) OT group [90 min saline +30 min OT (0.1 mg/kg)]+ MV (7 ml/kg, 120 breaths/min); 3) VILI group [90 min saline +30 min saline + MV (28 ml/kg, 60 breaths/min)]; 4) OT + VILI group [90 min saline +30 min OT (0.1 mg/kg) + MV (28 ml/kg, 60 breaths/min)]; 5) L-368,899 + OT + VILI group [90 min L-368,899 (5 mg/kg) + 30 min OT (0.1 mg/kg) + MV (28 ml/kg, 60 breaths/min)] following the experimental procedure as outlined below:

### 2.2.2 Part Two

Male C57BL/6 mice were randomly divided into four groups and all drugs were administered ip: 1) Control group [30 min normal saline + MV (7 ml/kg, 120 breaths/min)]; 2) VILI group [30 min normal saline +30 min MV (28 ml/kg, 60 breaths/min)]; 3) OT + VILI group [30 min OT (0.1 mg/kg) + MV (28 ml/kg, 60 breaths/min)], and 4) OT + nigericin + VILI group [30 min OT (0.1 mg/kg) + nigericin (5 mg/kg) + MV (28 ml/kg, 60 breaths/min)] following the experimental procedure as shown below:

All mice were anesthetized with an ip injection of 50 mg/kg sodium pentobarbital (Duque-Wilckens et al., 2018; An et al., 2019). After 4 h of ventilation treatment, all mice were sacrificed, and lung tissues and BALF were collected for further analysis.

### 2.3 Hematoxylin and Eosin Staining and Histopathological Assessment

The right upper lung lobe was collected, fixed with 4% paraformaldehyde, then sectioned and stained with hematoxylin and eosin (HE). The degree of lung inflammation in the alveolar tissue was assessed by two researchers who were blinded to the information concerning specimens using an optical microscope and assigning scores. Each tissue received the following scores based on the damage level: 0: normal tissue; 1: slight damage; 2: moderate damage; 3: severe damage; and 4: maximal damage (Vaschetto et al., 2008).

### 2.4 Measurement of Lung Wet/Dry Weight Ratio, Protein Concentrations, and MPO Activity in BALF

After 4 h of ventilation treatment, the lungs were lavaged with 0.3 ml PBS by tracheal intubation and pumped back after 3 s. The same operation was repeated three times in a total volume of 0.9 ml PBS, and the recovery rate was 90%. The lungs were blotted dry with filter paper, weighed to obtain the wet weight, and then were dried in an oven at 65°C for 48 h to obtain the dry weight. The lung wet/dry (W/D) ratio was calculated. Protein concentrations were determined using the BCA protein assay (ComWin Biotech, China). myeloperoxidase (MPO) activity, an indicator of the neutrophils and macrophages infiltration, was determined using test kits obtained from Nanjing Jiancheng Bioengineering Institute, China. The enzymatic activity was assessed by measuring the changes in the absorbance at 450 nm using a Varioskan Flash multifunction plate reader (Thermo Scientific, IL, United States).

### 2.5 Real-Time RT-PCR Analysis

The relative expression of different inflammation cytokines was measured using RT-qPCR. The total RNAs from fresh lung tissues were isolated using RNA Simple Total RNA kits (Aidlab Biotech, China), and then reverse transcription (RT) was performed on cDNA with Reverse Transcription Kit (Takara, Japan). Subsequently, qPCR was performed with the SYBR Green PCR Kit (Toyobo, Japan) and the Bioer Real-Time qPCR System (Bioer Technology, China). The 10-µl reaction system is comprised according to the report. The sequences of the specific primers Beijing Genomics Institute (Beijing, China) used in this study for quantitative real-time PCR are shown in Table 1. The expression levels of target mRNAs were normalized to actin.

**TABLE 1:** Primer sequences were used to detect the ASC, IL-1β, IL-6, NLRP3, GSDMD, IL-18, and Caspase-1 mRNAs with RT-qPCR.

Name	F (5′-3′)	R (5′-3′)
ASC	GTC​TTA​GGG​GCG​GAA​ACC​AA	CCG​CGG​TCA​CCT​TTT​ACT​CT
IL-1β	TGC​CAC​CTT​TTG​ACA​GTG​ATG	TGT​GCT​GCT​GCG​AGA​TTT​GA
IL-6	GTC​CTT​CCT​ACC​CCA​ATT​TCC​A	TAA​CGC​ACT​AGG​TTT​GCC​GA
NLRP3	CTC​GTC​ACC​ATG​GGT​TCT​GGT	AAC​GGA​CAC​TCG​TCA​TCT​TCA
GSDMD	GAT​CAA​GGA​GGT​AAG​CGG​CA	CAC​TCC​GGT​TCT​GGT​TCT​GG
IL-18	GCA​GTG​GTT​TTC​AGC​TGG​G	CAC​ACC​ACA​GGG​GAG​AAG​TG
Caspase-1	GGA​CCC​TCA​AGT​TTT​GCC​CT	AAC​TTG​AGC​TCC​AAC​CCT​CG

### 2.6 Measurement of Cytokine Levels in BALF

The levels of IL-1β, IL-6, and IL-18 in BALF were measured using mouse inflammatory cytokines ELISA kits (Wuhan Boster Biological Technology, China).

### 2.7 Western Blotting

Lung tissues were homogenized in the 4°C RIPA buffer (Beyotime Institute of Biotechnology, Shanghai, China) to obtain total protein. The protein concentration was determined using a BCA kit (ComWin Biotech, China). Subsequently, the protein samples were loaded and electrophoresed on SDS-polyacrylamide gels and were transferred onto membranes (Millipore, MA, United States). The membranes were blocked with a blocking solution (5% skim milk) for 1 h at room temperature and incubated with the following primary antibodies overnight at °C: anti-NLRP3 (dilution 1:1,000, Abcam 263899), anti-Caspase-1 (dilution 1:1,000, Abcam 108362), anti-TLR4 (dilution 1:1,000, Abcam 22048), anti-My-D88 (dilution 1:1,000, Abcam 2064), anti-NF-κB (dilution 1:1,000, Cell Signaling Technology 8242), anti-IL-1β (dilution 1:1,000, Abcam 9722), anti-IL-6 (dilution 1:1,000, Abcam 233706), anti-GSDMD (dilution 1:1,000, ab219800) and anti-β actin (dilution 1:4,000, Proteintech Group). The next day, all membranes were incubated with a secondary antibody conjugated to horseradish peroxidase at room temperature for 1 h. Finally, the membranes were imaged using enhanced chemiluminescence (ComWin Biotech, China) using a Bio-Rad ChemiDoc gel system.

### 2.8 Immunohistochemistry

The primary antibodies were anti-NLRP3 (Abcam 269899), and a VECTASTAIN Universal Quick Kit was also used. Secondary antibodies were Ready-to-Use Kit (Vector Laboratories, CA, United States). After being stained with 3, 3-diaminobenzidine (DAB), lung tissues were observed under a Nikon Eclipse 80i light microscope. We used ImageJ software to quantify intensities in all images.

### 2.9 Statistical Analysis

Data with normal distribution are expressed as mean ± standard deviation of the mean and the difference was evaluated using one-way ANOVA for statistical analysis among groups. Student’s *t*-test was performed for paired samples. For data that were not normally distributed, multiple comparisons were carried out using Kruskal-Wallis test. SPSS version 20.0 for Windows; SPSS Inc. Chicago, IL, United States) was used for analysis. *p* < 0.05 were considered statistically.

## 3 Results

### 3.1 The Protective Effect of OT on VILI

#### 3.1.1 OT Reduces Histopathological Lung Injury in the Mouse VILI Model

**FIGURE 2:**
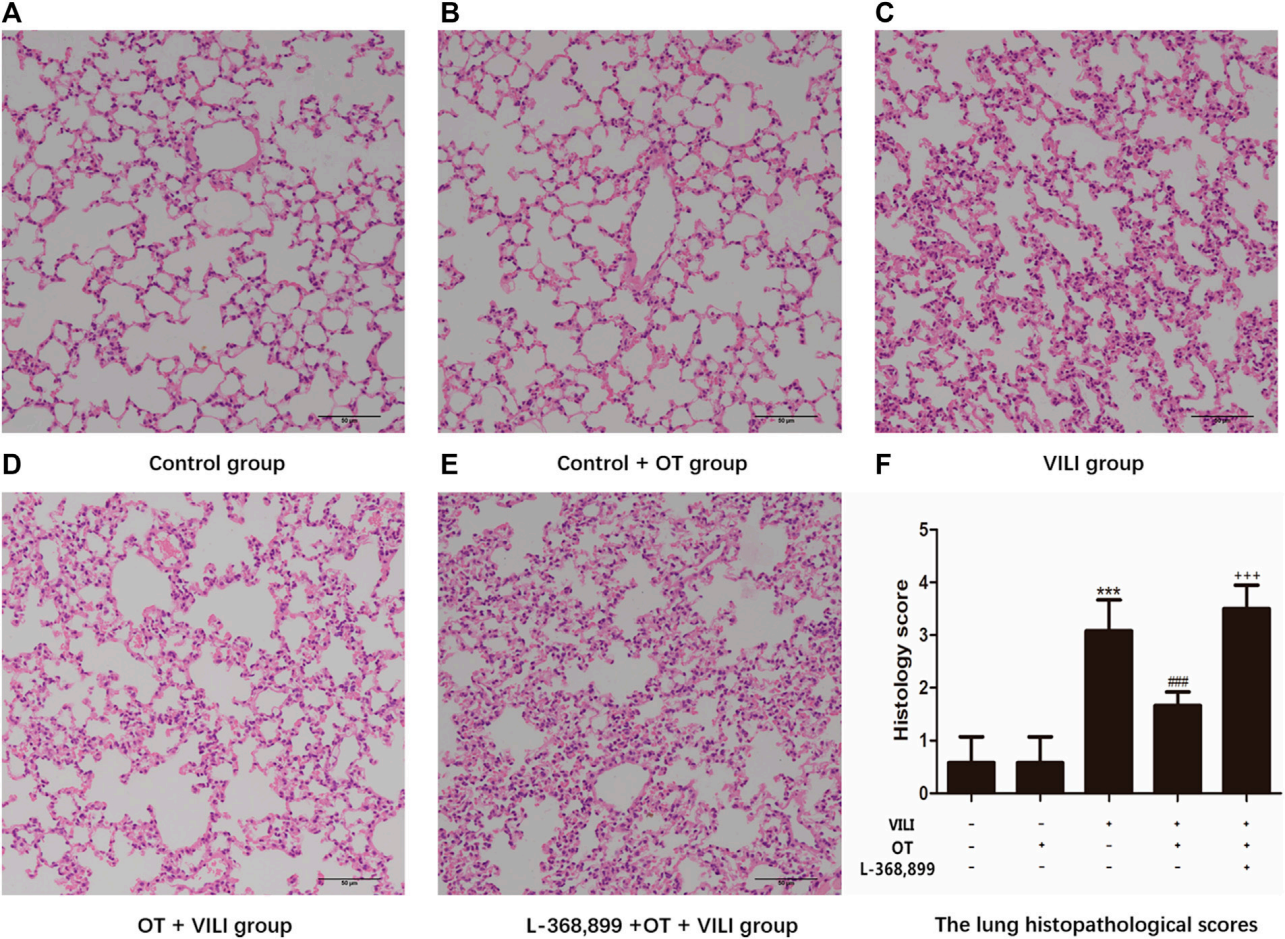
Oxytocin attenuates ventilation-induced lung injury-related pathological changes in the mouse model. Pathological changes in mouse lung tissues are observed using HE staining by light microscopy magnification ×200. **(A)** Control group. **(B)** Control + OT group. **(C)** VILI group. **(D)** OT + VILI group. **(E)** L-368,899 + OT + VILI group. **(F)** The lung histopathological scores are presented. The data are presented as the means ± SD (*n* = 5). ***p < 0.001 compared with the Control and Control + OT groups, ^###^ *p* < 0.001 compared with the VILI group, and ^+++^ *p* < 0.001 compared with the OT + VILI group. OT, oxytocin; SD, standard deviation; VILI, ventilation-induced lung injury.

We assessed pathological changes to evaluate the protective and anti-inflammatory effects of OT on VILI using HE staining. As shown in Figure 2, the Control group and Control + OT group exhibited an approximately typical structure (Figures 2A,B). The VILI group (Figure 2C) showed increased congestion, inflammatory cell infiltration, and degeneration in the lung. However, OT attenuated VILI-related pathological changes (Figure 2D). Furthermore, we presented histological lung injury scores in Figure 2F.

#### 3.1.2 OT Decreases the Lung W/D Ratio, Reduces Protein Concentration, and Inhibits MPO Activity in BALF

**FIGURE 3:**
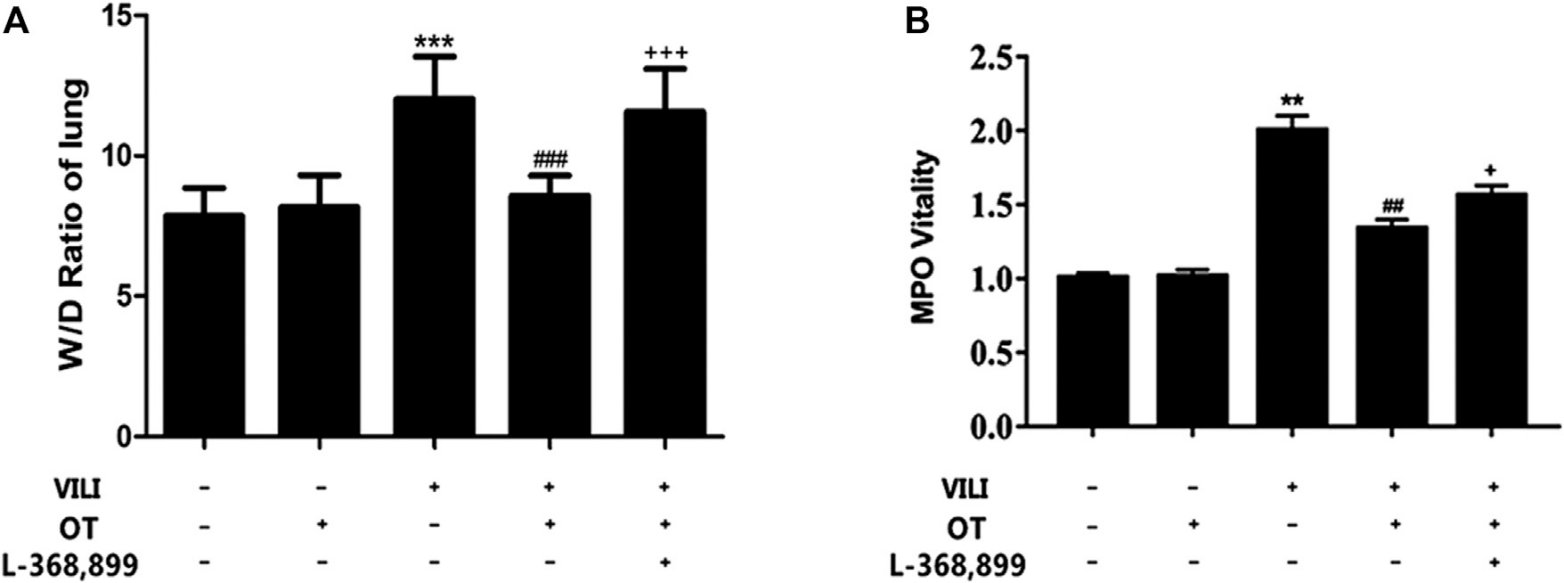
Oxytocin attenuated the lung wet/dry ratio and myeloperoxidase activity.Effects of OT on the lung W/D ratio **(A)** and MPO activity **(B)** are presented as the means ± SD. *p < 0.05, **p < 0.01 compared with the Control and Control + OT groups; ^#^ *p* < 0.05 and ^##^ *p* < 0.01 compared with the VILI group. ^+^ *p* < 0.05 and ^++^ *p* < 0.01 compared with the OT + VILI group (*n* = 5). MPO, myeloperoxidase; OT, oxytocin; SD, standard deviation; VILI, ventilation-induced lung injury.

We used W/D ratios to evaluate the pulmonary edema. Compared with the standard control, the W/D ratio of VILI mice was higher (Figure 3A). However, OT decreased the W/D ratio in lung tissues compared with that in the VILI group (Figure 3A). Moreover, OT reduced protein concentration in BALF of lung compared with that in the VILI group (Table 2). Meanwhile, the MPO activity in lung tissues of the VILI group was higher than that in the control group. However, compared with the VILI group, OT decreased MPO activity (Figure 3B).

**TABLE 2:** Effects of Oxytocin on protein permeability in BALF.

Group	Protein (mg/ml) in BALF
control	0.475 ± 0.013
control + OT	0.390 ± 0.010
VILI	0.808 ± 0.007**
OT + VILI	0.576 ± 0.009^##^
L-368899 + OT + VILI	0.703 ± 0.021++

Data are presented as the means ± standard deviations. **p < 0.01 compared with the Control and Control + OT groups; ##*p* < 0.01 compared with the VILI group. ^++^ *p* < 0.01 compared with the OT + VILI group.

#### 3.1.3 OT Decreases Production of Cytokines in Lung Tissue and BALF From the Mouse VILI Model

**FIGURE 4:**
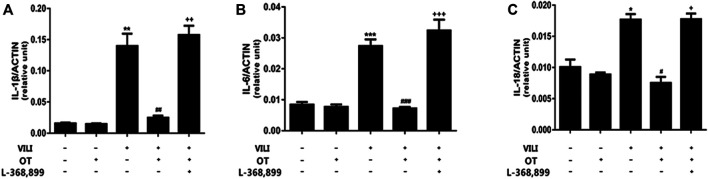
Oxytocin attenuates inflammatory cytokine gene expression in the lung tissues of ventilation-induced lung injury.Effects of OT on the expression of the IL-1β **(A)**, IL-6 **(B)**, and IL-18 **(C)** mRNAs in lung tissues are presented as the means ± SD (*n* = 4). *p < 0.05, **p < 0.01, and ***p < 0.001 compared with the Control and Control + OT groups; ^#^ *p* < 0.05, ^##^ *p* < 0.01, and ^###^ *p* < 0.001 compared with the VILI group. ^+^ *p* < 0.05, ++*p* < 0.01, and ^+++^ *p* < 0.001 compared with the OT + VILI group. OT, oxytocin; SD, standard deviation; VILI, ventilation-induced lung injury.

Inflammation is considered the primary response to injuries or infections. However, uncontrolled inflammatory repertoire may also lead to lung tissue damage (Morton et al., 2012; Szeto et al., 2017). We measured the levels of IL-1β, IL-6, and IL-18 in lung tissue and BALF using qPCR and ELISA to evaluate whether OT treatment decreased the inflammatory response in the mouse VILI model. As shown in Figure 4, the expression of IL-1β, IL-6, and IL-18 in lung tissue was increased in the VILI group compared with the control group (*p* < 0.05). However, OT decreased the levels of IL-1β, IL-6, and IL-18 in lung tissues compared with the VILI group.

#### 3.1.4 OT Reduces OTR Expression in the Lung Tissues of the Mouse VILI Model

**FIGURE 5:**
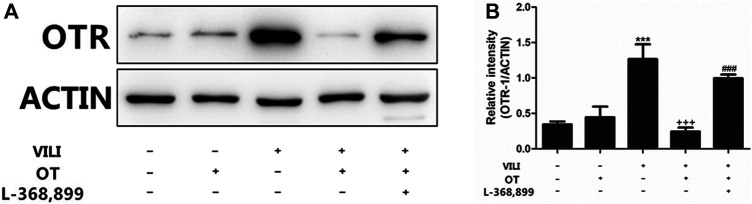
Effects of oxytocin on the expression of OTR protein.OT reduces the expression of OTR protein in the mouse VILI model. Western blot was performed to evaluate the protein expression of OTR in lung tissue **(A)**. The levels of OTR protein expression were quantified by measuring band intensities and displayed as fold increase relative to ACTIN **(B)**. The values are presented as means ± SD (*n* = 5). ***p < 0.001 compared with the Control and Control + OT groups, ^###^ *p* < 0.001 compared with the VILI group, and ^+++^ *p* < 0.001 compared with the OT + VILI group. OT, oxytocin; SD, standard deviation; VILI, ventilation-induced lung injury.

In this study, we analyzed OTR expression to investigate the underlying mechanism of OT. As shown in Figure 5, the expression of the OTR protein in the VILI group was increased compared with that in the control group. However, compared with the VILI group, OT reduced the expression of the OTR protein in the OT + VILI group.

#### 3.1.5 OT Administration Inhibits TLR4/My-D88/NF-κB Activation in the Mouse VILI Model

**FIGURE 6:**
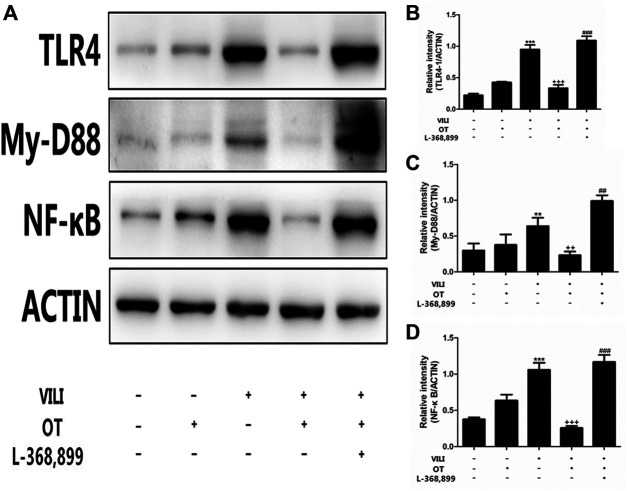
Effects of oxytocin on the expression of TLR4, My-D88, and NF-κB protein.OT administration inhibited TLR4-mediated My-D88/NF-κB activation in the mouse VILI model. Western blot was performed to evaluate the protein expression of TLR4, My-D88, and NF-κB in lung tissue exposed to mechanical ventilation **(A)**. The levels of TLR4 **(B)**, My-D88 **(C)**, NF-κB **(D)** protein expression were quantified by measuring band intensities and displayed as fold increase relative to ACTIN. The values presented are the means ± SD (*n* = 5). **p < 0.01 and ***p < 0.001 compared with the Control and Control + OT groups, ^##^ *p* < 0.01 and *p* < 0.001 compared with the VILI group, and ^++^ *p* < 0.01 and ^+++^ *p* < 0.001 compared with the OT + VILI group. OT, oxytocin; SD, standard deviation; VILI, ventilation-induced lung injury.

Next, we used western blotting to investigate the TLR4/My-D88/NF-κB pathway activation after VILI by determining protein levels (Figure 6). Compared with the control and control + OT groups, TLR4 levels were increased in the VILI group (Figure 6). Meanwhile, compared with those in the VILI model group, OT inhibited TLR4, My-D88, and NF-κB expression.

#### 3.1.6 OT Administration Inhibits NLRP3/Caspase-1 Expression in the VILI Mouse Model

**FIGURE 7:**
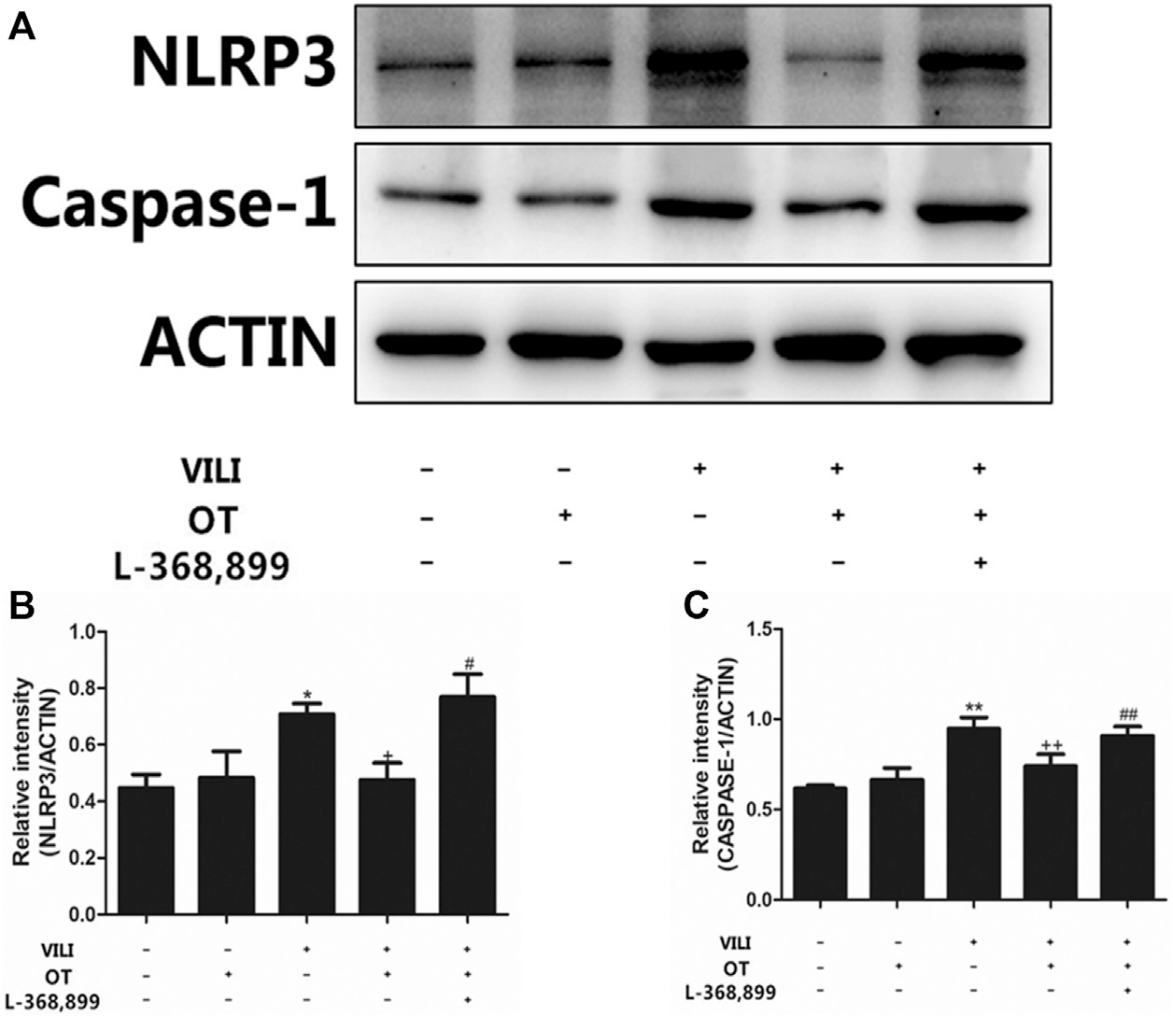
Effects of oxytocin on the expression of NLRP3 and Caspase-1.OT administration inhibited NLRP3 and Caspase-1 expression in the mouse VILI model. Western blot was performed to evaluate the protein expression of NLRP3 and Caspase-1 in lung tissue exposed to mechanical ventilation **(A)**. The levels of NLRP3 **(B)** and Caspase-1 **(C)** protein expression were quantified by measuring band intensities and displayed as fold increase relative to ACTIN. The values presented are the means ± SD (*n* = 5). *p < 0.05 and **p < 0.01 compared with the Control and Control + OT groups, ^#^ *p* < 0.05 and ^##^ *p* < 0.01 compared with the VILI group, and ^+^ *p* < 0.05 and ^+++^ *p* < 0.01 compared with the OT + VILI group. OT, oxytocin; SD, standard deviation; VILI, ventilation-induced lung injury.

In this study, we used western blotting to investigate the expression of NLRP3/caspase-1. As shown in Figure 7, compared with the control group and the control + OT group, MV increased NLRP3 and caspase-1 proteins expression (*p* < 0.05). However, compared with the VILI group, OT decreased the expression of these proteins (Figure 7).

#### 3.1.7 L-368,899, an Antagonist of the Oxytocin Receptor, Could Weaken the OT’s Effects

As shown in Figure 2E, compared with the OT + VILI group, we found that L-368,899 could aggravate histopathological changes in the lungs. Moreover, L-368,899 administration increased the W/D ratio and MPO activity compared with the OT + VILI group (Figures 3A,B). Furthermore, L-368,899 increased inflammatory cytokine levels compared with the OT + VILI group (Figure 4). Compared with the OT + VILI group, L-368,899 administration increased the expression of the OTR, TLR4/My-D88/NF-κB and NLRP3/caspase-1 (Figures 5−7).

### 3.2 Nigericin has Potentially Partial Effect on OT’s Anti-Inflammation

#### 3.2.1 Effects of OT on the Expression of NLRP3, Caspase-1, IL-1β, and GSDMD in Lung Tissues of VILI Mice Treated With and Without Nigericin

**FIGURE 8:**
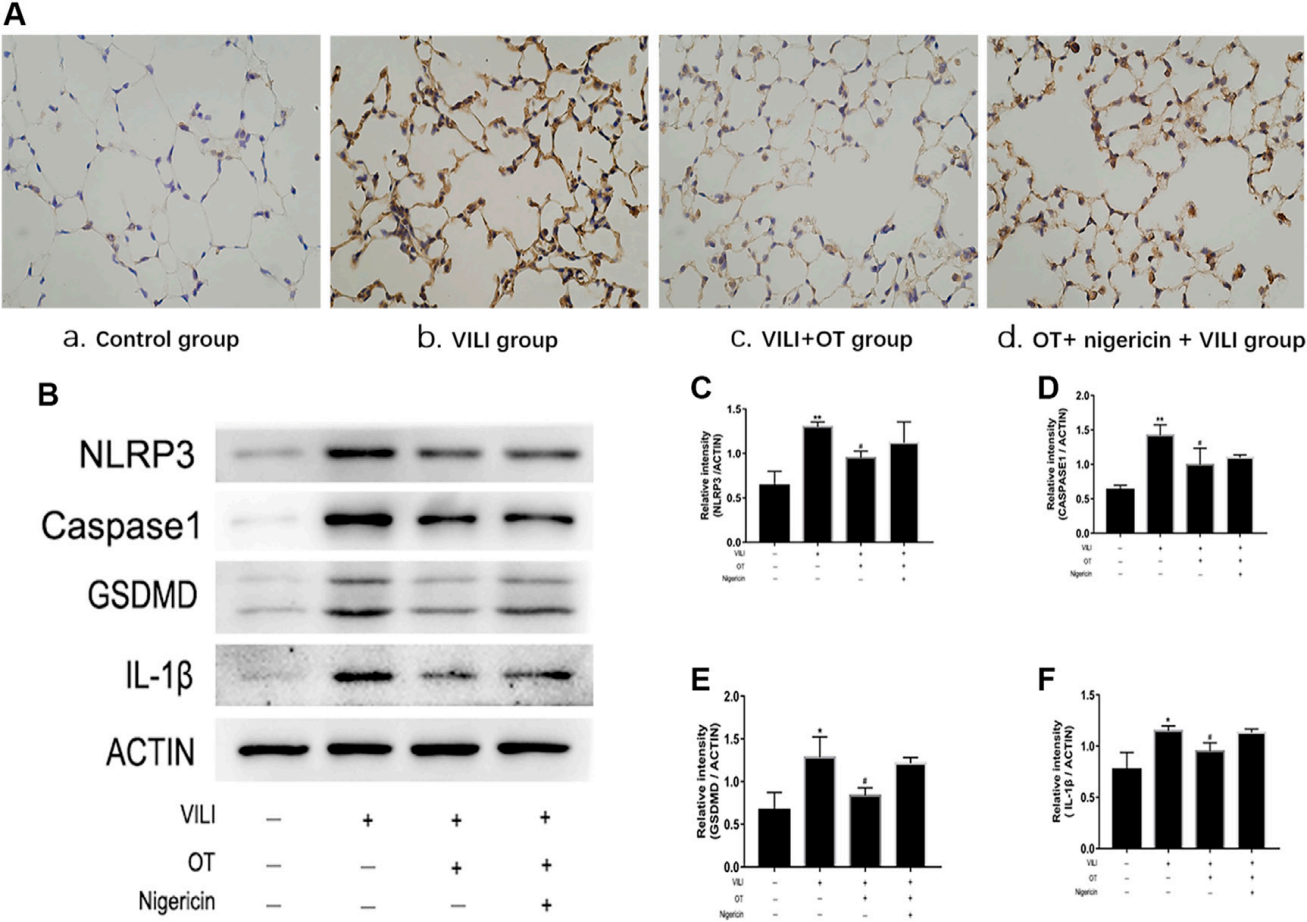
Effects of oxytocin on the expression of NLRP3, Caspase-1, GSDMD, and IL-1β in lung tissues from VILI mice treated with and without Nigericin. **(A)** Immunohistochemistry (IHC) staining of NLRP3 of different groups: 1) Control group, 2) VILI group, 3) VILI + OT group, 4) OT + nigericin + VILI group, and **(B)** Western blot analysis of NLRP3, Caspase-1, GSDMD and IL-1β levels were quantified. The levels of NLRP3 **(C)**, Caspase-1 **(D)**, GSDMD **(E)**, and IL-1β **(F)** protein expression were quantified by measuring band intensities and displayed as fold increase relative to ACTIN. The values presented are the means ± SD (*n* = 5). *p < 0.05 and **p < 0.01 compared with the control group and ^##^ *p* < 0.05 and ^##^ *p* < 0.01 compared with the VILI group. OT, oxytocin; SD, standard deviation; VILI, ventilation-induced lung injury.

Immunohistochemistry and western blotting with antibodies against NLRP3, caspase-1, IL-1β, and GSDMD were performed to determine whether NLRP3-mediated pyroptosis in response to OT. As shown in Figures 8A,B, elevated NLRP3 levels were observed in the mouse VILI model; OT (0.1 mg/kg) treatment decreased the expression of NLRP3, caspase-1, IL-1β, and GSDMD proteins in the mouse VILI model. However, compared with the OT treatment alone or control group, the OT plus nigericin treatment increased the expression levels of NLRP3, caspase-1, IL-1β, and GSDMD in VILI mice. In addition, GSDMD is the critical substrate and the direct executioner of pyroptosis. Therefore, our results indicate that the anti-pyroptosis effect of OT could be partially mediated through the activation of NLRP3.

#### 3.2.2 OT Reduces Inflammatory Cytokine Gene Expression in Lung Tissues of VILI Mice

**FIGURE 9:**
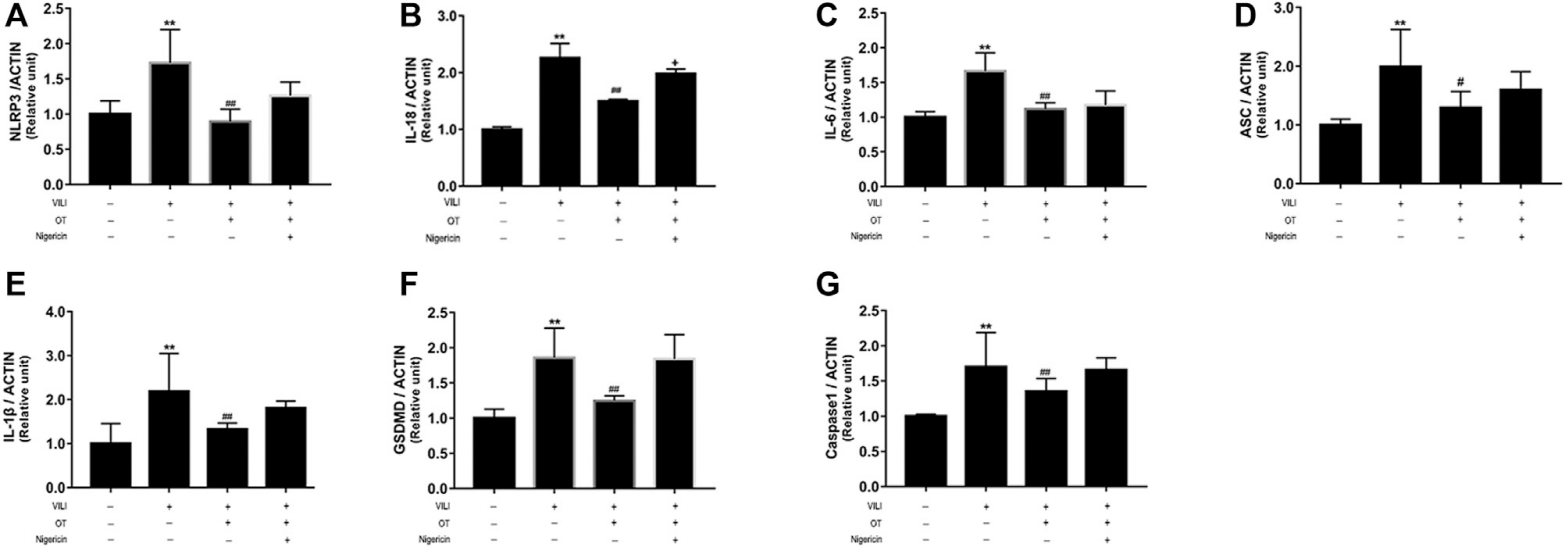
Effects of oxytocin on the expression of NLRP3, IL-18, IL-6, ASC, IL-1β, GSDMD, and Caspase-1 in lung tissues from VILI mice treated with or without Nigericin.RT-qPCR is performed to evaluate the expression of the NLRP3 **(A)**, IL-18 **(B)**, IL-6 **(C)**, ASC **(D)**, IL-1β **(E)**, GSDMD **(F)**, and Caspase-1 **(G)** mRNAs in lung tissues from mice exposed to mechanical ventilation in the presence or absence of Nigericin. Data are presented as the means ± SD (*n* = 5). *p < 0.05, and **p < 0.01 compared with the Control group; ^#^ *p* < 0.05 and ^##^ *p* < 0.01 compared with the VILI group. OT, oxytocin; SD, standard deviation; VILI, ventilation-induced lung injury.

We use real-time quantitative PCR (qPCR) to determine cytokine expression in the lung tissues. Higher levels of NLRP3, IL-18, IL-6, ASC, IL-1β, GSDMD, and caspase-1 mRNAs were detected in the VILI group than in the control group (Figure 9). However, OT administration alleviated the levels of the NLRP3, ASC, IL-18, IL-6, IL-1β, GSDMD, and Caspase-1 mRNAs in the VILI group. Compared with expression in the OT group or control group, VILI treated with OT plus nigericin showed increased expression levels of GSDMD. Therefore, our results indicate that NLRP3 partially mediates the anti-pyroptotic effect of OT.

#### 3.2.3 Effects of OT on the Concentration of IL-1β, IL-18, IL-6 in BALF in VILI Mice Treated With and Without Nigericin

**FIGURE 10:**
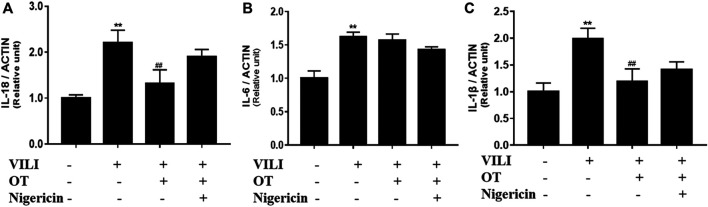
Effects of oxytocin on cytokine concentrations of IL-18, IL-6, and IL-1β in the BALF from VILI mice treated with or without Nigericin.The expression of the IL-1β **(A)**, IL-6 **(B)**, and IL-18 **(C)** in the BALF from mice VILI model. The data are presented as the means ± SD (*n* = 5). *p < 0.05, and **p < 0.01 compared with the Control group; ^#^ *p* < 0.05 and ^##^ *p* < 0.01 compared with the VILI group. OT, oxytocin; SD, standard deviation; VILI, ventilation-induced lung injury.

The levels of inflammatory cytokines including IL-1β, IL-6, and IL-18 in the BALF, were detected using ELISA kits. As shown in Figure 10, increased levels IL-1β, IL-6, and IL-18 were observed in the mouse VILI model compared with the control group. However, the expression level of these cytokines was markedly decreased in the OT-treated group compared with the VILI group, while this effect was partly reversed by nigericin (*p* < 0.05). Previous studies indicated that NLRP3 activation mediated the process of pro-IL-18 and pro-IL-1β into their mature form. Therefore, our results indicate that NLRP3 could partially mediates the release of IL-1β and IL-18 in the VILI mice model.

## 4 Discussion

OT, a naturally occurring 9-amino acid neuropeptide hormone, exerts anti-ovarian tumor effects both *in vivo* and *in vitro* due to its antiproliferative properties (Morita et al., 2004; Mankarious et al., 2016; Ji et al., 2018). Moreover, it has been shown to reduce inflammation in multiple preclinical disease models (Jankowski et al., 2010; Yeniel et al., 2014; Xiaomei et al., 2017). As shown in our previous study, oxytocin protected against LPS-induced lung injury by blocking the activation of the TLR4/NLRP3/NF-κB signaling pathway. Inflammation plays a crucial role in and contributes to the initiation and progression of VILI; however, the effect of OT on VILI has not been explored, and the direct downstream pathway of NLRP3 has not been elucidated yet (An et al., 2019). In the present study, we identified that TLR4-and NLRP3/GSDMD-mediated pyroptosis signaling were activated in VILI mice. Our data also indicated that GSDMD, the critical factor in the process of pyroptosis, was closely correlated with VILI. In addition, NLRP3 signaling might be involved in the mechanism of GSDMD-related pyroptosis in VILI. In summary, the significant finding in our study revealed the protective effect of OT on modulating the TLR4-and NLRP3/GSDMD-mediated inflammatory pathway in a mouse VILI model.

After activation, the NLRP3 inflammasome recognizes many types of dangerous molecules and pathogens and participates in the immune response, ultimately causing various immune or metabolic diseases. Nigericin, uric acid crystals, or LPS cause NLRP3 to bind to the adaptor protein ASC, which oligomerizes to recruit procaspase-1 in caspase-1(Wu et al., 2013; Humayun et al., 2019). Eventually, pro-IL-18 and pro-IL-1β are processed into their mature form, resulting in lung injury. In our present study, MV markedly upregulated the expression of TLR4/My-D88/NF-κB and NLRP3/caspase-1, suggesting that TLR4-and the NLRP3-mediated pathways played essential roles in VILI. Moreover, OT administration significantly attenuated the MV-induced upregulation of TLR4/My-D88/NF-κB and NLRP3/caspase-1. Meanwhile, our study also indicated that OT partially reduced VILI by inhibiting the activation of the TLR4 and the NLRP3 mediated inflammasome pathways (Duque-Wilckens et al., 2018; Gamer and Bü chel, 2012).

Recent studies indicated that GSDMD was one of the crucial proteins involved in caspase-1-mediated pyroptosis and controlled the release of IL-1β in the bone marrow macrophages of the mouse (He et al., 2015; Shi et al., 2015). In addition, pyroptosis was triggered by cleaving of GSDMD to generate an N-terminal cleavage product (GSDMD-NT). However, whether GSDMD also contributed to the development of pyroptosis in VILI has seldom been reported. Our data indicated the increased expression of GSDMD in VILI lung tissues. The OT administration significantly decreased the expression of GSDMD, accompanied by the inhibition of pyroptosis and the release of IL-18, IL-1β, and IL-6 in the VILI model. Moreover, the NLRP3 agonist nigericin partially reversed the protective effect of OT on VILI, indicating that OT potentially protected against VILI through NLRP3-mediated inflammatory pathway. In summary, our results show that GSDMD, which plays a crucial role in inducing pyroptosis, maybe a potential candidate for developing novel targeted therapies in VILI.

Inflammation is the major response to injuries or infections. However, prolongation and uncontrolled inflammation, which is characterize as cytokine production, may lead to tissue damage (Chanez et al., 2007; Ji et al., 2018). Previous studies indicated that MV may activate maturation and release of inflammatory cytokines production such as IL-1β, IL-18, or IL-6. Our results also indicated that MV markedly increased the production of IL-1β, IL-18, and IL-6 in BALF and the expression of IL-1β, IL-6, and IL-18 in lung tissues of VILI mice. Previous studies showed that OT modulated the OTR expression (Morita et al., 2004; Mankarious et al., 2016; An et al., 2019.). In addition, our previous work indicated that OT reduced lung injuries and the production of inflammatory cytokines in LPS-induced lung injury (An et al., 2019). Moreover, previous studies indicated that both MV and nigericin could induce the maturation and release of IL-1β and IL-18 through the activation NLRP3 inflammasome (Jankowski et al., 2010; Xiaomei et al., 2017). In the present study, the OT administration significantly decreased the expression of IL-1β and IL-18 at both the protein and mRNA levels. Thus, it was indicated by our findings that OT exerts potential anti-inflammatory effects as a treatment for VILI by inhibiting the production of IL-1β, IL-18, which is mediated by NLRP3.

## 5 Conclusion

In summary, our report provides the first evidence of a protective effect of OT on VILI through the TLR4-and NLRP3/GSDMD-mediated signaling pathways *in vivo*. Our findings will facilitate further investigations of a potential target for the treatment of VILI and other inflammatory diseases.

## Data Availability

The raw data supporting the conclusions of this article will be made available by the authors, without undue reservation.
