# Supplementary material for: Protective Effect of Oxytocin on Ventilator-Induced Lung Injury Through NLRP3-Mediated Pathways
Source: Front Pharmacol. 2021 Oct 18;12:722907. doi: 10.3389/fphar.2021.722907 (PMC8558354; doi:10.3389/fphar.2021.722907)

**Supplemental figure S1：**Full scan of the original blots of cropped images shown in Figure 4 A.

Lane1: marker. Lane2: Control group; Lane3: Control + OT group; Lane4: VILI group; Lane5: OT + VILI group; Lane6: L-368,899 + OT + VILI group; Lane7: marker.

ACTIN OTR












**Supplemental figure S2**：Full scan of the original blots of cropped images shown in Figure 5 A.

Lane1: marker. Lane2: Control group; Lane3: Control + OT group; Lane4: VILI group; Lane5: OT + VILI group; Lane6: L-368,899 + OT + VILI group; Lane7: marker.

ACTIN TLR4












My-D88 NF-κB












**Supplemental figure S3**：Full scan of the original blots of cropped images shown in Figure 6 A.

Lane1: marker. Lane2: Control group; Lane3: Control + OT group; Lane4: VILI group; Lane5: OT + VILI group; Lane6: L-368,899 + OT + VILI group; Lane7: marker.

ACTIN NLRP3












Caspase-1







**Supplemental figure S4：**Full scan of the original blots of cropped images shown in Figure 7 B.

Figure 7: Full scan of the original blots of cropped images shown in figure7B

Lane1: Marker/

Lane2: control

Lane3: NS+VILI group

Lane4: OT+VILI group

Lane5: OT+VILI+Nigericin group

ACTIN NLRP3


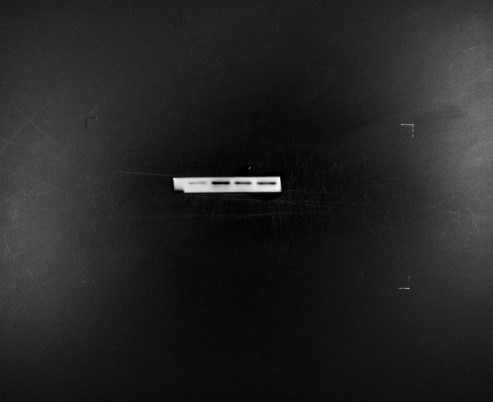

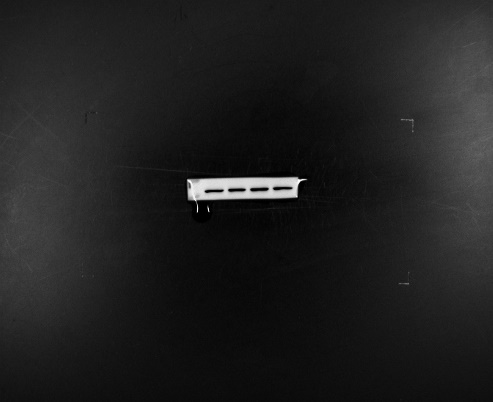


Caspase-1 GSDMD




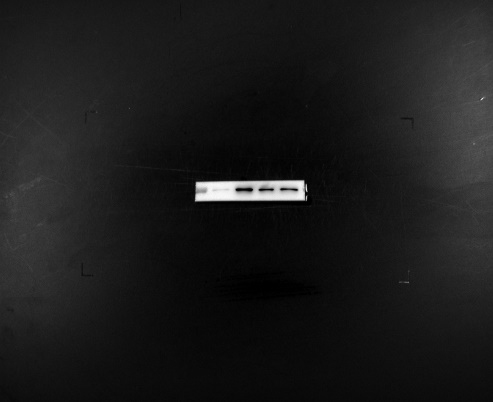


IL-1β


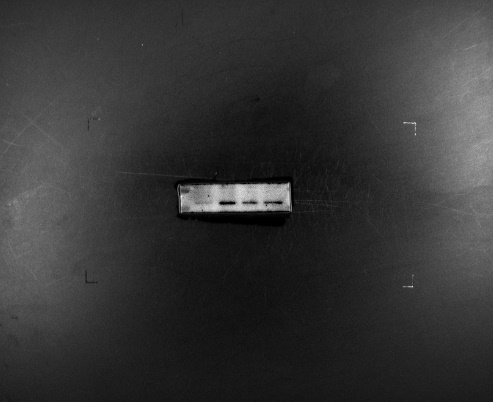

Supplement: Supplementary file 1 [file DataSheet1.docx]
